# The antimycobacterial and healing effect of sorafenib through pro-apoptotic and immunomodulatory activities

**DOI:** 10.1128/spectrum.01657-25

**Published:** 2026-01-26

**Authors:** Raju S. Rajmani, Nidhi Rani, Avadhesha Surolia

**Affiliations:** 1 Molecular Biophysics Unit, Indian Institute of Science, Bangalore, India; 2 Dr. Reddy's Institute of Life Sciences 231940 , Hyderabad, India; LSU Health Shreveport, Shreveport, Louisiana, USA

**Keywords:** *Mycobacterium tuberculosis *(*Mtb*), sorafenib (SRB), apoptosis, necrosis, immunomodulation

## Abstract

Tuberculosis is caused by the bacterium *Mycobacterium tuberculosis (Mtb*). Emergence of drug resistance in *Mtb* requires continuous enrichment of anti-tubercular medication. Inclusion of host-directed therapies holds considerable promise in this context. Sorafenib (SRB) is a multi-kinase inhibitor targeting VEGF receptor kinase, Raf, MEK, and extracellular signal-regulated kinase (ERK) signaling cascade to treat several types of cancer, including hepatocellular carcinoma. We have previously established that SRB allosterically inhibits ornithine acetyltransferase (MtArgJ), an essential enzyme in the arginine biosynthesis pathway of *Mtb*, thereby limiting bacterial growth in culture at a minimum inhibitory concentration of 10 µg/mL. The current work focuses on how SRB at the dose of 30 mg/kg body wt inhibits the pathogenicity and survival of bacteria in a preclinical mouse model of tuberculosis by inducing pro-apoptotic and immunomodulatory mechanisms in the host. We observed that SRB treatment promotes apoptosis in *Mtb*-infected and -uninfected THP-1 cells, human monocyte-derived macrophages. Concomitantly, SRB treatment reduces infection-associated necrosis in the *Mtb*-infected THP-1 cells. We further noted the upregulated expression of pro-apoptotic proteins during SRB treatment in preclinical mouse models. In addition, we investigated the expression of pro- and anti-inflammatory cytokines and immunomodulation in lung tissues treated with SRB. Interestingly, SRB treatment increased the number of arginase 1-positive macrophages, which are reckoned to enhance tissue healing. In conclusion, our research discloses that SRB is helpful in both lowering the tubercular burden and accelerating recovery of damaged tissue by harnessing the host immune response.

**IMPORTANCE:**

Host-directed therapies hold considerable promise for treating drug-resistant *Mycobacterium tuberculosis* (*Mtb*). In this context, the induction of apoptotic and immunomodulatory responses in the host by sorafenib (SRB) is demonstrated here to compromise the survival and pathogenic potential of *Mtb* in a preclinical mouse model of TB and in *Mtb*-infected and -uninfected THP-1 cells. Concurrently, the infection-associated necrosis in the *Mtb*-infected THP-1 cells is also reduced. Furthermore, arginase 1-positive macrophages, which are known to enhance tissue healing, are increased in SRB-treated groups. Thus, SRB treatment not only lowers the tubercular load but also aids in healing damaged tissues by leveraging the host immunity.

## INTRODUCTION

*Mycobacterium tuberculosis* (*Mtb*) is one of the most successful and deadly pathogens afflicting humans. *Mtb,* as an infectious agent, is the leading cause of death worldwide, which even now infects millions of people each year (1). The fatality linked to *Mtb* is further exacerbated by the emergence of drug-resistant strains, co-infection with HIV, and socioeconomic conditions in underdeveloped countries (2–6). Thus, it is imperative to not only enrich the anti-tubercular pipeline but also endeavor to shorten its treatment. Developing anti-tuberculosis (anti-TB) medications by host-directed approaches that target the pathogen’s intracellular survival constitutes a promising approach to counter drug-resistant strains while minimizing the possibility of the appearance of drug-resistant strains (7–9). However, several FDA-approved repurposed drugs are undergoing clinical studies at different stages to treat TB infections, including some that aim to shorten the duration of its treatment (10). Sorafenib (SRB), an FDA-approved drug, is a multikinase inhibitor for treating several types of cancers. We have previously demonstrated that SRB allosterically inhibits ornithine acetyltransferase (MtArgJ), an essential enzyme in the arginine biosynthesis pathway of *Mtb*, thereby decreasing bacterial growth both *in vitro* and *ex vivo* settings (11). However, it was also reported that SRB induced TB reactivation in a TB patient (12). The current work focuses on the immunomodulatory and pro-apoptotic mechanisms that SRB recruits in the host to inhibit the pathogenicity and survival of the bacteria. Apoptosis is a highly regulated and coordinated process of programmed cell death in which *Mtb* and the cell components of dying macrophages released from the cell as apoptotic bodies, encased in cytoplasmic membranes, are phagocytosed in the host (13), which not only limits the intracellular development of bacilli but also activates dendritic cells (DCs) and sets off strong adaptive immunological responses (14, 15). *Mtb*, however, has developed several ways to interfere with macrophage responses, including modulation of apoptosis to flourish therein (16). Therefore, it is essential to curtail the mechanisms behind *Mtb’s* manipulation of macrophage apoptosis for the development of an efficacious treatment. In this study, we found that SRB administration induces apoptosis in both the infected and uninfected THP-1 cells. Furthermore, SRB diminishes the infection-related necrosis in human monocyte-derived macrophages infected with *Mtb*. We also investigated how SRB therapy affected the expression of pro-apoptotic proteins in a preclinical mouse model. The expression of pro- and anti-inflammatory cytokines and immunomodulation was also investigated in lung tissues treated with SRB. Interestingly, SRB therapy improved tissue repair by increasing the quantity of arginase 1 (Arg-1)-positive macrophages. In summary, our study showed that SRB helps to reduce the tubercular burden, concurrently healing the injured tissues.

## MATERIALS AND METHODS

### Bacterial culture

Middlebrook 7H9 broth media (Difco) supplemented with 10% albumin-dextrose-catalase (Becton, Dickinson), 0.4% glycerol, and 0.05% Tween 80 were used to cultivate *Mtb* (H37Rv strain), a virulent laboratory strain of *Mtb*, American Type Culture Collection. The *Mtb* cultures were grown to mid-log phase (OD_600_ 0.4–0.7) for experiments.

### Animal husbandry

A required number of 6- to 8-week-old female BALB/c mice were procured from the Central Animal Facility at IISc, Bangalore. Before being infected, the animals were allowed to acclimatize for 2 weeks at the ABSL-3 laboratory.

### Apo-BrdU (TUNEL) staining

TUNEL staining was performed using an Apo-BrdU TUNEL Assay Kit (cat. no. A23210), Invitrogen/ Thermo Fisher Scientific, and according to the manufacturer’s protocol. Briefly, THP-1 cells were harvested using EDTA, taking care to keep all non-adherent cells. Cells were washed with PBS and fixed overnight in 4% paraformaldehyde in PBS. Cells were washed twice in PBS, 1 mL cold ethanol was added, and cells were stored at −22°C overnight. Cells were washed twice with wash buffer, and BrdUTP was incorporated into DNA nicks using TdT enzyme for 60 min at 37°C. The cells were then washed twice with rinse buffer and incubated with FITC-anti-BrdU antibody for 30 min at room temperature (RT). The cells were washed again with rinse buffer, fixed with 4% paraformaldehyde, and analyzed using a FACS ARIA flow cytometer. FACS data were analyzed by FACS Diva software.

### PE annexin V and 7-AAD staining

For analyzing live, early apoptotic, late apoptotic, and necrotic populations, we used PE Annexin V Apoptosis Detection Kit I (cat. no. 559763), BD Pharmingen. The staining was performed according to the manufacturer’s protocol. Briefly, THP-1 cells were harvested using EDTA, taking care to keep all non-adherent cells. Then the cells were washed twice with cold PBS and then resuspended in 1× binding buffer at a concentration of 1 × 10^6^ cells/mL. After that, we used 5 µL of PE annexin V and 5 µL of 7-AAD for staining the cells and incubated for 15 min at RT (25°C) in the dark. Finally, we added 400µL of 1× binding buffer to the incubated cells, and the samples were acquired on a BD FACSAria Fusion flow cytometer (BD Biosciences, San Jose, CA).

### Study design for chronic and acute infection models

#### Chronic model

For the chronic model of infection, BALB/c mice of 6 to 8 weeks old (≈20 g) were infected via aerosol through a Madison aerosol generation instrument calibrated to deliver 100 colony-forming units (CFU). Mice were housed for 4 weeks to establish chronic infection of *Mtb* in the lungs. After 4 weeks post-infection, the mice were randomly grouped (*n* = 5) as vehicle control (H37Rv-infected), SRB alone treated, isoniazid (INH) alone treated, or SRB + INH in combination treated. Following 8 weeks of drug administration (SRB: 5 weekly doses of 30 mg/kg body wt via I/P route, and INH: 25 mg/kg body wt via drinking water daily), the mice were humanely sacrificed to assess the bacterial load in their lungs.

#### Acute model

To assess the bactericidal activity of SRB, we established an acute model of infection. In this model of infection, 6- to 8-week-old (≈20 g) female BALB/c mice were infected with ≈500 bacilli (≈500 CFU) of H37Rv, and after 1 week post-infection (≈10^3^ CFU), mice were randomly grouped as the SRB alone treated group and the vehicle (H37Rv-infected) control group (*n* = 6). After 4 weeks of treatment with SRB 30 mg/kg body wt via I/P (five dosages per week), the mice were humanely sacrificed to enumerate the bacterial burden in their lungs and spleens.

#### Infection of animals

On the day of infection, bacterial cultures (H37Rv) in the mid-log phase (OD_600_ 0.4–0.7) were passed through several needle gauges ranging from lower to higher (23G, 26G, and 30G) to create a single-cell suspension. The OD_600_ of the culture was then measured. The Madison Aerosol Chamber was used to aerosol-infect the animals. To determine the infection dose, five infected animals were sacrificed after 24 h post-infection, and lungs were extracted, homogenized, and plated on 7H11 agar plates for CFU count. At the proper point in the study design, the remaining animals were humanely killed by cervical dislocation. To determine CFU, the lungs and spleens were removed and weighed. The remaining tissues were homogenized in 2 mL of PBS, and the lung homogenate at the proper dilution was plated on 7H11 agar media supplemented with oleic albumin dextrose catalase and polymyxin B, amphotericin B, nalidixic acid, trimethoprim, and azlocillin for the counting of CFU. A portion of the right upper lobe of the lungs was preserved in 10% neutral buffered formalin for histology. Every experiment was carried out in IISC Bangalore’s BSL-3 lab.

#### Histological analysis and evaluation

Following animal sacrifice, the right upper lobe of the lung tissues was kept until it was prepared for histological analysis by infusing it with 10% neutral buffered formalin. Sections of 4 µm thickness from formalin-fixed and paraffin-embedded tissues were cut onto glass slides and stained with hematoxylin and eosin (H&E) for histological assessment and imaging at the time of tissue processing. Granulomas and granuloma regions with certain pathologies were counted.

#### Granuloma scores

Individual granulomas were manually counted and observed in order to calculate the granuloma score. We created a scientific approach to use pathological markers to score the development of granulomas (9, 17). Necrotic granulomas received a score of 5, non-necrotic granulomas received a score of 2.5, and fibrotic granulomas received a score of 1. The total granuloma scores were calculated by adding these scores together. The ratio of the granuloma’s total area to the section’s total area was used to determine the percentage of organ area occupied (Table 1).

**TABLE 1:** Methodology of the granuloma scores

	No. of granulomas without necrosis × 2.5	No. of granulomas with necrosis × 5	No. of granulomas with fibrosis × 1	Total granuloma score
Example	5 × 2.5 = 12.5	1 × 5 = 5	0 × 1 = 0	17.5

#### Histopathology scores

The histopathological evaluation was carried out using the histopathology scoring system for the lung tissues. We created a scientific method that modified Mitchison’s virulence scoring system to consider the type of immune cell infiltration, the number and locations of granulomas, necrosis, and alveolar consolidation. Histopathology scores ranged from 0 to 4 (6, 18). Severe pathology received a score of 4, moderate pathology received a score of 3, minimum pathology received a score of 2, minor pathology received a score of 1, and no pathology received a score of 0.

### Preparation of tissue lysate and western blotting

Lung tissue lysate was prepared in RIPA (10×-0.5 M NaCl, 0.5M EDTA, pH 8.0, 1 M Tris, NP-40, 10% sodium deoxycholate, 10% SDS) buffer containing 10% protease inhibitor cocktail (Roche) for 30 min on ice. Total protein was estimated using the Bradford (Bio-Rad) method of protein estimation. Protein samples were subjected to 12% SDS polyacrylamide gel electrophoresis and then were transferred onto 0.45 μm PVDF membrane (18 V, 2 h). The membrane was blocked using 5% skim milk in TBST (Tris-buffered saline containing 0.1% Tween-20) for 1 h at RT and subsequently probed with appropriate primary antibody (Table 2) overnight at 4°C. Following washing in TBST, the blot was probed with specific HRP-conjugated secondary antibody for 1 h at RT. β-Actin was used as the loading control. The membrane was developed using ECL (Advansta), and images were captured using ChemiDoc (GE Healthcare). All densitometric analysis was performed using ImageJ software.

**TABLE 2:** List of primary antibodies used in the present investigation for Western blotting

Antibody name	Catalog number
Phospho-Akt (Ser473) (D9E) XP rabbit mAb	Cat #4060 (Cell Signaling Technology)
Phospho-mTOR (Ser2448) antibody	Cat #2971 (Cell Signaling Technology)
mTOR antibody	Cat #2972 (Cell Signaling Technology)
Phospho-p44/42 MAPK (Erk1/2) (Thr202/Tyr204) antibody	Cat #9101 (Cell Signaling Technology)
Stat1 antibody	Cat #9172 (Cell Signaling Technology)
Cleaved caspase-3 (Asp175) (5A1E) rabbit mAb	Cat #9664 (Cell Signaling Technology)
Cleaved caspase-7 (Asp198) antibody	Cat #9491 (Cell Signaling Technology)
β-Actin antibody	Cat #4967 (Cell Signaling Technology)

### RNA isolation and quantitative real-time PCR

The lung tissues were harvested in RNAlater (Thermo Fisher Scientific) and stored at −80°C. Total RNA was isolated by homogenizing the tissues in TRIzol (Takara) and incubated with chloroform for phase separation. Total RNA was precipitated from the aqueous layer by treatment with isopropanol and 75% ethanol wash. Quantification of RNA was performed using a NanoDrop (Thermo Fisher Scientific). The First Strand cDNA synthesis kit (Applied Biological Materials Inc.) was used to convert an equivalent amount of RNA into cDNA. The cDNA was utilized for quantitative real-time PCR analysis of the relevant genes using SYBR Green (Thermo Fisher Scientific). β-Actin was used as the internal control gene. The qPCRs were set up in a 384-well plate with two replicates for each sample. Primer pairs used for expression analyses are provided below (Table 3).

**TABLE 3:** List of primers used for gene expression analysis

Genes	Forward primer (5′−3′)	Reverse primer (5′−3′)
Mouse β-actin	CACTGTCGAGTCGCGTC	TCATCCATGGCGAACTGGTG
Mouse IFN-γ	CACGGCACAGTCATTGAAAG	GCTGATGGCCTGATTGTCTT
Mouse IL-2	TTGTGCTCCTTGTCAACAGC	CTGGGGAGTTTCAGGTTCCT
Mouse IL-10	GAGCAGGTGAAGAGTGATTT	AGGAGTTGTTTCCGTTAGC
Mouse FoxP3	CCCATCCCCAGGAGTCTT	ACCATGACTAGGGGCACTGTA
Mouse TGF-β	CCCTATATTTGGAGCCTG	GTTGGTTGTAGAGGGCAA

### Processing of lungs for immune cell isolation

The lungs were removed aseptically, minced, and digested in a 2% FBS/DMEM solution with 0.2 mg/mL liberase and 0.1 mg/mL of DNase (Roche). The mixture was then incubated for 30 min at 37°C while being shaken. Subsequently, the pre-digested lung samples underwent processing in a Miltenyi Biotec gentle MACS dissociator, following the usual lung dissociation technique. After the lung material was processed, it was passed through a 70 µm cell strainer. The filtered samples were then centrifuged at 1,600 rpm for 5 min at 4°C, and the supernatant was discarded. Next, the pellet containing the red blood cells (RBCs) was lysed with freshly made RBC lysis buffer and allowed to sit at RT for 5 to 10 min. To counteract the lysis buffer’s effects, 10 mL of PBS was added to each sample. The lung cell suspensions were then treated for 5 min on ice with Fc block (BD Biosciences), after which the cells were counted and stained for cell surface markers with a cocktail of antibodies and incubated in the dark at RT for 30 min. For the intracellular antigens, BD Biosciences’ fix and perm buffer was used to fix and permeabilize the cells. The samples were then treated with antibodies in accordance with the BD Biosciences protocol. Samples were acquired on a BD FACSAria Fusion flow cytometer (BD Biosciences, San Jose, CA). The antibodies used in the present investigation are listed in Table 4. Additionally, we acknowledge that the Materials and Methods section in the current study was also used in our earlier studies (9, 19) and therefore has some identical descriptions.

**TABLE 4:** List of antibodies used in the present investigation for flow cytometry

Antibodies	Catalog no. and company
Alexa Fluor 700 mouse anti-mouse CD45.1	# 561235, BD Pharmingen
PE-Cy7 hamster anti-mouse CD11c	# 558079, BD Pharmingen
BV480 rat anti-CD11b	# 566117, BD Horizon
BV510 rat anti-mouse Ly-6G	# 740157, BD OptiBuild
BV605 rat anti-mouse Ly-6C	# 563011, BD Horizon
Alexa Fluor 488 rat anti-mouse F4/80-like receptor	# 564227, BD Pharmingen
Arginase 1 monoclonal antibody (A1exF5), eFluor 450	# 48-3697-82, eBioscience

### Analysis

The flow cytometry data were analyzed using BD FACSDiva version 8.0.1 and FlowJo 10.8.0 software (BD Biosciences). The gating sequence included the following order: live (SSC-A^+^ FSC-A^+^), FSC-H vs FSC-A to exclude doublets (singlet gate), SSC-A^+^CD45^+^ (leukocytes), and so on.

### Statistical analysis

Statistical analyses were conducted using GraphPad Prism software (version 8.0), and values are presented as mean ± SD. Statistical significance between two experimental groups was determined by a two-tailed, unpaired Student’s *t*-test (*P < 0.05, **P < 0.01, ***P < 0.001, ****P < 0.0001, and *P* > 0.5; n.s., not significant). For more than two experimental groups, the statistical significance was determined by one-way ANOVA as well as an appropriate post-test. *P*-values are represented as ns, not significant, *P < 0.05, **P < 0.01, ***P < 0.001, ****P < 0.0001.

## RESULTS

This study demonstrates that by triggering apoptosis within the host, SRB therapy dramatically reduces the bacterial burden in both acute and chronic infection models of TB. It also discusses the possible immunomodulatory impact of SRB in lowering the bacterial burden and in the repair of pulmonary pathology in cohorts treated with SRB.

### SRB treatment induced apoptosis and inhibited necrosis in *Mtb*-infected and -uninfected THP-1 cell line

**Fig 1:**
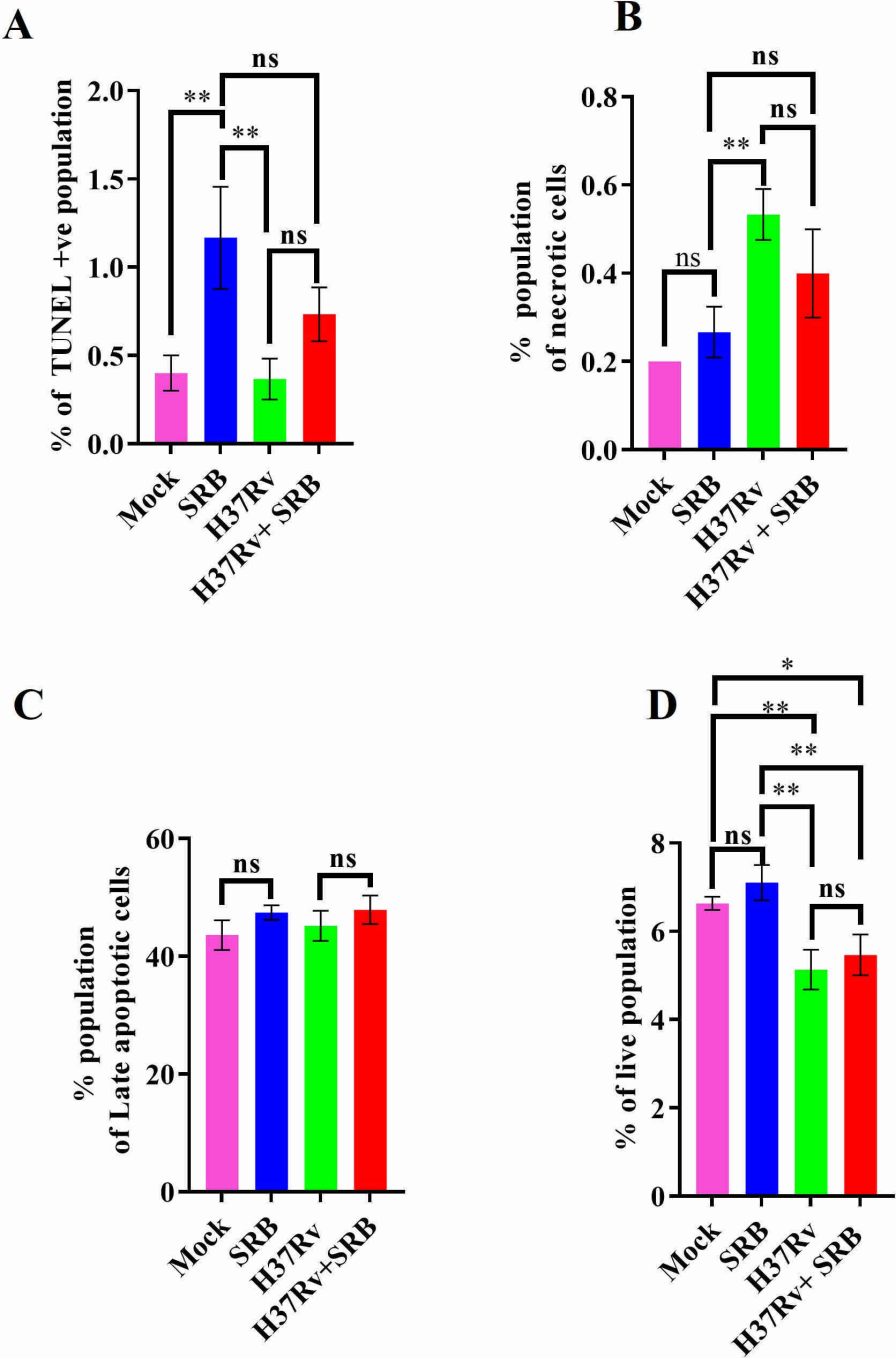
Assessment of apoptosis and necrosis during *Mtb* infection and SRB treatment in THP-1 macrophages. THP-1 macrophages were infected with *Mtb* strain H37Rv (MOI 10) and treated with SRB (10 µM) for 48 h. (**A**) Apoptosis was determined by staining with Apo-BrdU-FITC and the bar diagram showing percentage of TUNELpositive population, respectively, in *Mtb*-infected and SRB-treated THP-1 macrophages. (**B–D**) Determination of necrotic (annexin-V−/7AAD+), late apoptotic (annexin-V+/7AAD+), and live (annexin-V−/7AAD−) populations, respectively, in *Mtb*-infected and SRB-treated THP-1 macrophages after 48 h of infection (10 MOI) and SRB treatment. Data information: data are obtained from three biological replicates and presented as mean ± SD. Statistical significance between experimental groups was determined by one-way ANOVA with Tukey’s multiple comparison test. *P*-values determined and represented as ns, non-significant, *P < 0.05, and **P < 0.01.

We have previously documented that SRB allosterically inhibits ornithine acetyltransferase (MtArgJ), an essential enzyme in the arginine biosynthesis pathway of *Mtb*, thereby decreasing bacterial growth in both *in vitro* and *ex vivo* settings (11). In the current study, we report additional beneficial aspects of SRB in managing TB. We used the *Mtb* H37Rv strain (10 MOI) to infect the human monocytic cell line THP-1 following PMA treatment. SRB (10 µM concentrations) was then administered to the *Mtb*-infected and -uninfected THP-1 cells for a duration of 48 h. Subsequently, the cells were harvested, processed, and stained with ApoBrdU-TUNEL, PE annexin V, and 7-AAD, and run in FACS to analyze the apoptotic and necrotic populations of THP-1 cells. We observed that SRB treatment promotes apoptosis in *Mtb*-infected and -uninfected THP-1 cell lines. Additionally, we observed that *Mtb*-uninfected THP-1 cells had a higher apoptotic population than *Mtb-*infected cells following SRB treatment (Fig. 1A). These findings show that *Mtb* curtails apoptosis while SRB increases it, indicating that *Mtb* and SRB have antagonistic effects on apoptotic processes in macrophages. Subsequently, SRB treatment reduces the infection-associated necrosis in the *Mtb*-infected human monocyte-derived macrophages. Here, we also found that SRB treatment considerably reduces the necrotic population in *Mtb*-uninfected THP-1 cells compared to *Mtb*-infected THP-1 cells. Additionally, SRB administration to *Mtb*-infected THP-1 cells results in an insignificant reduction in the necrotic population (Fig. 1B). These findings showed that SRB inhibits necrosis while *Mtb* encourages it. Thus, *Mtb* and SRB also work antagonistically toward necrosis-related activities. The results thus highlight the ability of SRB in enhancing apoptosis concomitantly with diminution of necrosis in THP-1 cells infected with *Mtb*. Which, in turn, is detrimental in the dissemination of *Mtb,* as evident from diminished bacterial load in spleens of SRB-treated animals. Furthermore, we also observed that the nonsignificant increase in the populations of late apoptotic cells in SRB-treated groups, either infected with H37Rv or without it (Fig. 1C). Furthermore, we analyzed the percent of live population among the groups and found a nonsignificant increase in the populations of live cells in SRB-treated groups in contrast to mock control and infected with H37Rv, respectively. However, we observed a significant decrease in live cell population in H37Rv-infected groups with or without SRB treatment as compared to the mock control and mock treated with SRB (1D). Representative dot plots showing apoptotic, necrotic, late apoptotic, and live population in SRB-treated and H37Rv-infected control groups are presented in Fig. S1 at https://github.com/rajmani57/Supplementary-file_antimycobacterial-and-immunomodulatory-activities-of-Sorafenib.

### SRB treatment reduced the *Mtb* burden and tubercular granulomas in the lungs of *Mtb*-infected mice in the chronic model of infection and treatment

**Fig 2:**
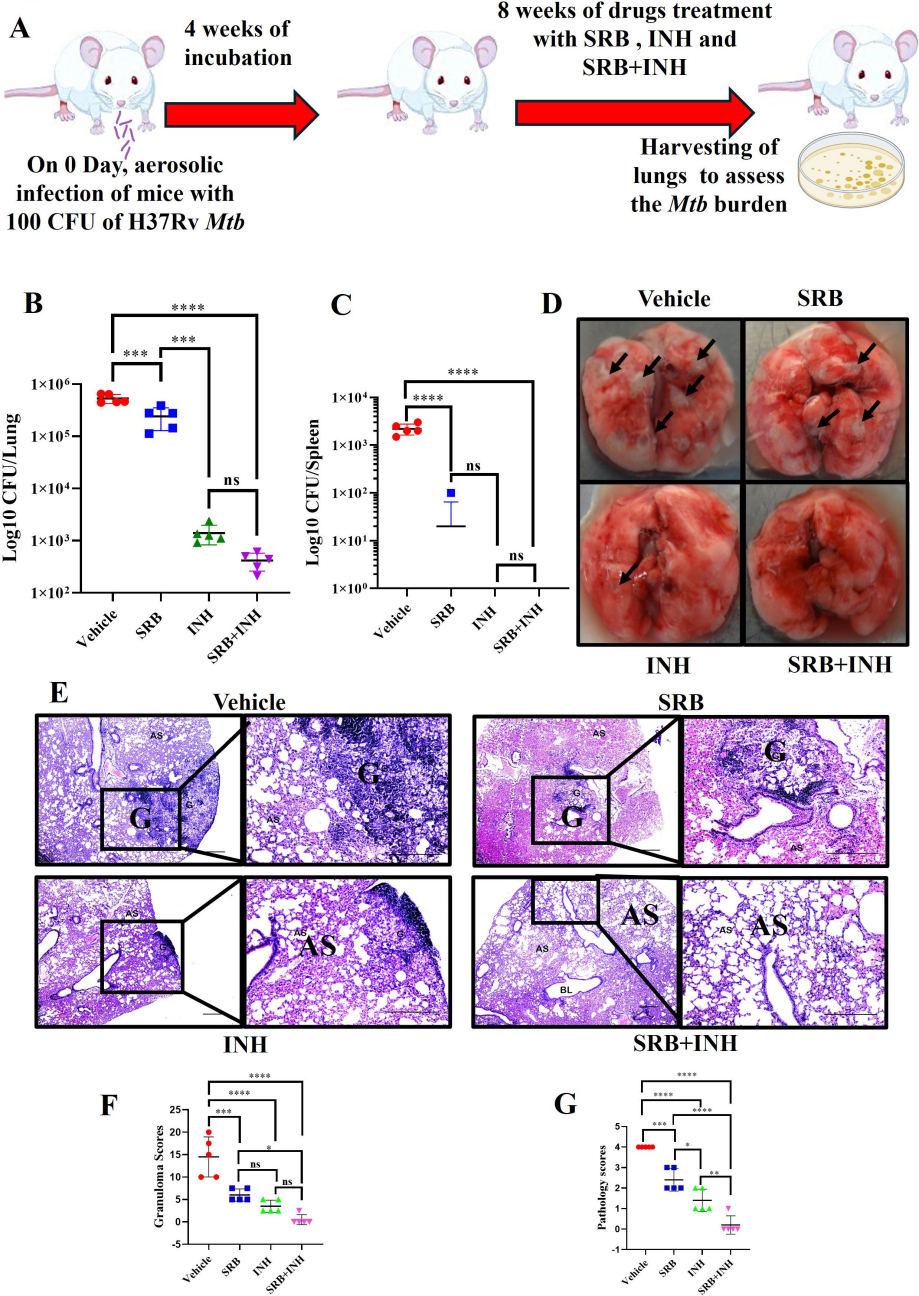
SRB treatment significantly reduces the *Mtb* burden in the lungs of *Mtb-*infected mice. (**A**) Schematic of *Mtb* infection and drug treatment in BALB/c mice. (**B**) Bacterial count in lungs at 8 weeks post-treatment (*n* = 5). (**C**) Bacterial count in spleens at 8 weeks post-treatment. For Fig. 2B and C, the data were collected from five mice in each cohort and are presented as mean ± SD in log10 CFU/lung and log10 CFU/spleen, respectively, and the statistical significance between experimental groups was determined by one-way ANOVA with Tukey’s multiple comparison test. *P*-values determined and represented as ns, non-significant; ***P < 0.001 and ****P < 0.0001. (**D**) Representative gross morphology of lungs from vehicle (H37Rv-infected) control, SRB-, INH-, and SRB+INH-treated mice (8 weeks post-treatment). The black arrows on lungs correspond to tubercular granuloma. (**E**) H&E-stained representative images of histopathology of the lungs from the vehicle (H37Rv-infected) control, SRB-, INH-, and SRB+INH-treated groups. G, granuloma; AS, alveolar space (scale bar, 100 µm). Histopathological analysis, granuloma score (**F**), and histopathology score (**G**) for vehicle (H37Rv-infected) control, SRB-, INH-, and SRB+INH-treated groups in the chronic infection and treatment models. The data were collected from five mice (*n* = 5) in each cohort and are presented as mean ± SD, and the statistical significance between experimental groups was determined by one-way ANOVA with Tukey’s multiple comparison test. *P*-values are determined and represented as ns, non-significant; *P < 0.05; **P < 0.01; ***P < 0.001; and ****P < 0.0001.

Following up on our previous research, we conducted a toxicity experiment of SRB in BALB/c mice and found no toxicity of SRB at the dose of 30 mg/kg body wt, administered weekly for 5 days per week for 4 weeks continuously through oral route (see Fig. S2 at https://github.com/rajmani57/Supplementary-file_antimycobacterial-and-immunomodulatory-activities-of-Sorafenib). Then we examined the effectiveness of SRB alone and in conjunction with isoniazid (INH) in a preclinical model of chronic *Mtb* infection and therapy in mice. We observed that SRB treatment significantly reduced the *Mtb* burden in the lungs of mice in the chronic model of *Mtb* infection. Furthermore, we noted that SRB in combination with INH worked better than INH alone and showed maximum diminution of *Mtb* burden in infected mice after 8 weeks of treatment. The differences in bacterial load between vehicle (H37Rv-infected) control and SRB-treated groups were 0.344 logs after 8 weeks of treatment. Similarly, the bacterial load difference between INH-alone-treated and SRB+INH in combination-treated groups was 0.527 logs after 8 weeks of treatment (Fig. 2B). In addition, dissemination of bacteria was observed in five of five vehicle control groups, with bacterial loads of 2 × 10^3^ CFU in the spleens. However, in one of five SRB-treated mice, we observed bacterial dissemination with only 100 CFU in the spleen. In INH-alone- and SRB+INH-treated cohorts, complete sterilization of bacterial burdens was observed (Fig. 2C). Gross pathology of lungs in the vehicle control group and SRB-alone-, INH-alone-, and SRB+INH combination-treated groups after 8 weeks of treatment, respectively, was found to be compatible with the CFU counts (Fig. 2D). We observed severe pathology with multiple small and large granulomas, alveolar consolidation, and infiltration of immune cells in the lungs of vehicle control. Similarly, moderate to mild pathology in the lungs of SRB-alone and mild pathology in INH-alone-treated groups of mice lungs after 8 weeks of treatment was observed. In SRB+INH combination treatment groups, we observed minimal to no pathology with large areas of normal alveolar space (Fig. 2E). Granuloma scores and histopathology scores in the vehicle control, SRB alone, INH alone, and SRB+INH in combination therapy groups were found to be in agreement with bacterial infection and treatment, respectively (Fig. 2F and G). Therefore, SRB and INH work better in combination to lower the bacterial burden. Together, these findings show that SRB has antimycobacterial properties in *Mtb*-infected mice and that combining SRB with INH considerably reduces the bacterial burden in chronic animal models of TB infection.

### SRB treatment regulates apoptotic pathways through Akt and mTOR signaling

**Fig 3:**
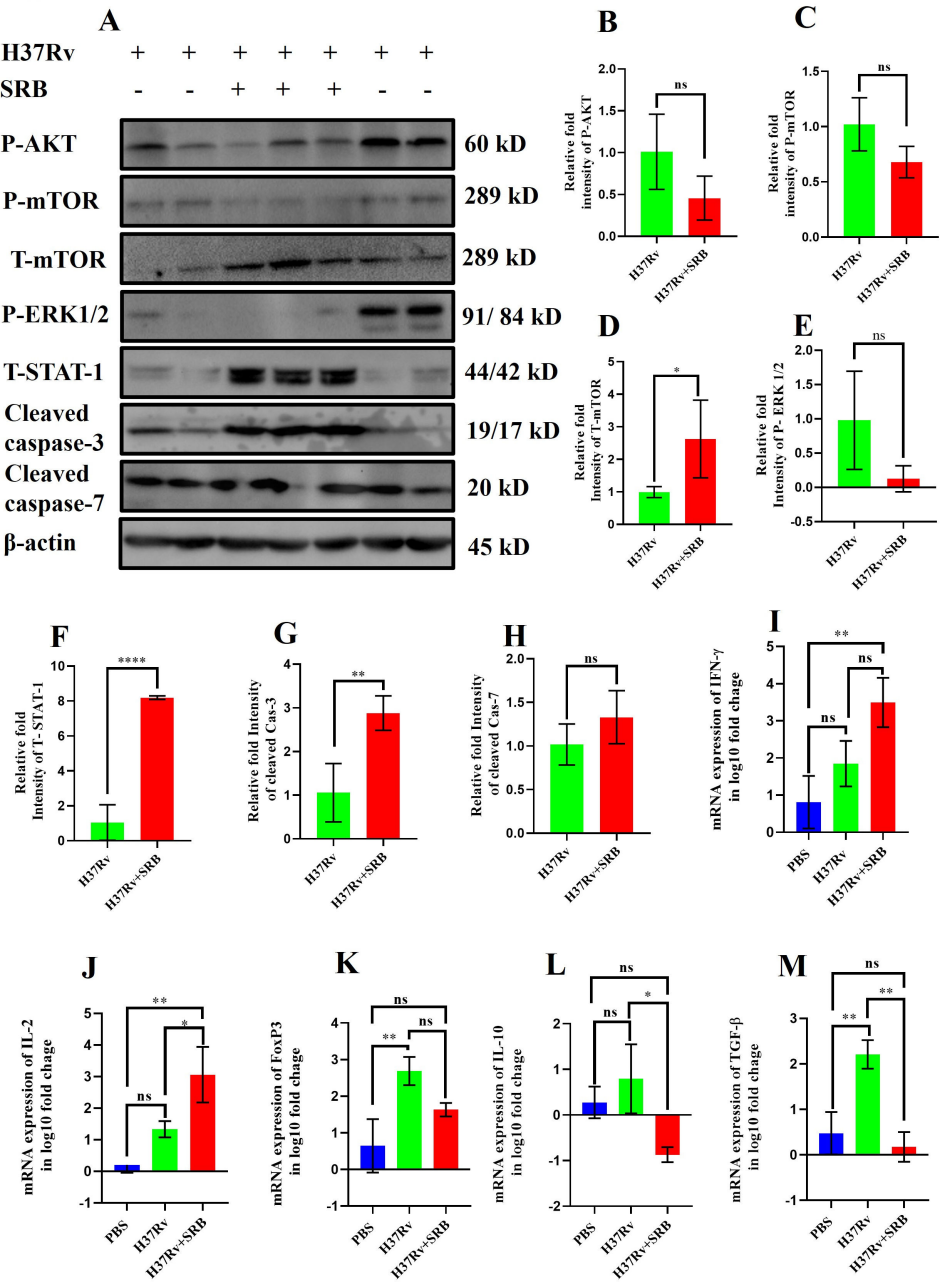
Western blotting to examine how SRB affected proteins and signaling molecules linked to apoptosis. (**A**) Western blots depicting the expression levels of P-AKT, P-mTOR, T-mTOR, P-ERK1/2, T-STAT-1, cleaved caspase-3, cleaved caspase-7, and β-actin, respectively. (**B**) Densitometric plot depicting the band intensities of P-AKT over β-actin. (**C**) Densitometric plot depicting the band intensities of P-mTOR over β-actin. (**D**) Densitometric plot depicting the band intensities of T-mTOR over β-actin. (**E**) Densitometric plot depicting the band intensities of P-ERK1/2 over β-actin. (**F**) Densitometric plot depicting the band intensities of T-STAT-1 over β-actin. (**G**) Densitometric plot depicting the band intensities of cleaved caspase-3 over β-actin. (**H**) Densitometric plot depicting the band intensities of cleaved caspase-7 over β-actin. For Fig. 3B through H, the data were collected from four mice of H37Rv-infected untreated control and from three mice in the SRB-treated cohort, and are presented as mean ± SD. The statistical significance between experimental groups was determined by an unpaired Student’s *t*-test (*P < 0.05, **P < 0.01, ****P < 0.0001, and n.s., not significant. (**I**) mRNA expression level of IFN-γ in log10 fold change over β-actin. (**J**) mRNA expression level of IL-2 in log10 fold change over β-actin. (**K**) mRNA expression level of FoxP3 in log10 fold change over β-actin. (**L**) mRNA expression level of IL-10 in log10 fold change over β-actin. (**M**) mRNA expression level of TGF-β in log10 fold change over β-actin. Data information: for panels I through M, the data were obtained from three biological replicates and presented as mean ± SD, and the statistical significance between experimental groups was determined by one-way ANOVA with Tukey’s multiple comparison test. *P*-values determined and represented as ns, non-significant; *P < 0.05; and **P < 0.01.

Various proteins of the cell signaling pathways, such as Akt (V-akt murine thymoma viral oncogenes homolog 1), mTORC1 (mammalian target of rapamycin complex 1), ERK1/2, and STAT-1 (signal transducer and activator of transcription 1), are crucial for cell proliferation and cell death. The PI3K-Akt pathway prevents apoptosis and increases cell survival, and the activation of the PI3K-Akt pathway prevents cell death by phosphorylating downstream targets that decrease apoptotic proteins. On the other hand, blocking this process may increase the likelihood that cells may undergo apoptosis. The mTORC1 activation promotes cell survival and growth by suppressing death pathways, while its inhibition can trigger apoptosis. In contrast, mTORC1 phosphorylation can have opposing effects on apoptosis. Apart from these, ERK1/2 activation is a key component of the signaling pathway that drives cell proliferation and survival, and through the activation of pro-apoptotic genes and proteins such as caspases, STAT-1 facilitates apoptosis. We have done Western blotting to examine how SRB affected proteins and signaling molecules linked to apoptosis. We noted a decrease in p-AKT, p-mTORC, and p-ERK1/2, respectively, in SRB-treated groups in contrast to only H37Rv-infected control groups (Fig. 3A through C and E). SRB treatment reduced the phosphorylation of mTOR at the Ser2488 position (Fig. 3A, C, and D) and decreased the phosphorylation of Akt-1 at the Ser473 location (Fig. 3A and B). Furthermore, the SRB-treated groups exhibited a noticeably greater amount of total STAT-1 proteins (Fig. 3A and F), and consequently, an increased level of cleaved caspase-3 and cleaved caspase-7 (Fig. 3A, G, and H) was observed in contrast to the H37Rv-infected control. In addition, the SRB-treated group had considerably higher levels of pro-inflammatory IFN-γ and IL-2 mRNA (Fig. 3I and J) and lower levels of FoxP3, IL-10, and TGF-β mRNA (Fig. 3K through M), respectively. This is consistent with earlier studies that showed that individuals with active TB had higher levels of FoxP3+ Treg cells and immunosuppressive cytokines such as IL-10 and TGF-β (20, 21). In summary, SRB therapy promotes unphosphorylated STAT-1 while blocking the phosphorylation of Akt, mTOR, and ERK1/2, respectively. These mechanisms ultimately combine to activate caspase-3, which triggers apoptosis.

### SRB treatment reduced the *Mtb* burden in the acute model of infection and treatment

**Fig 4:**
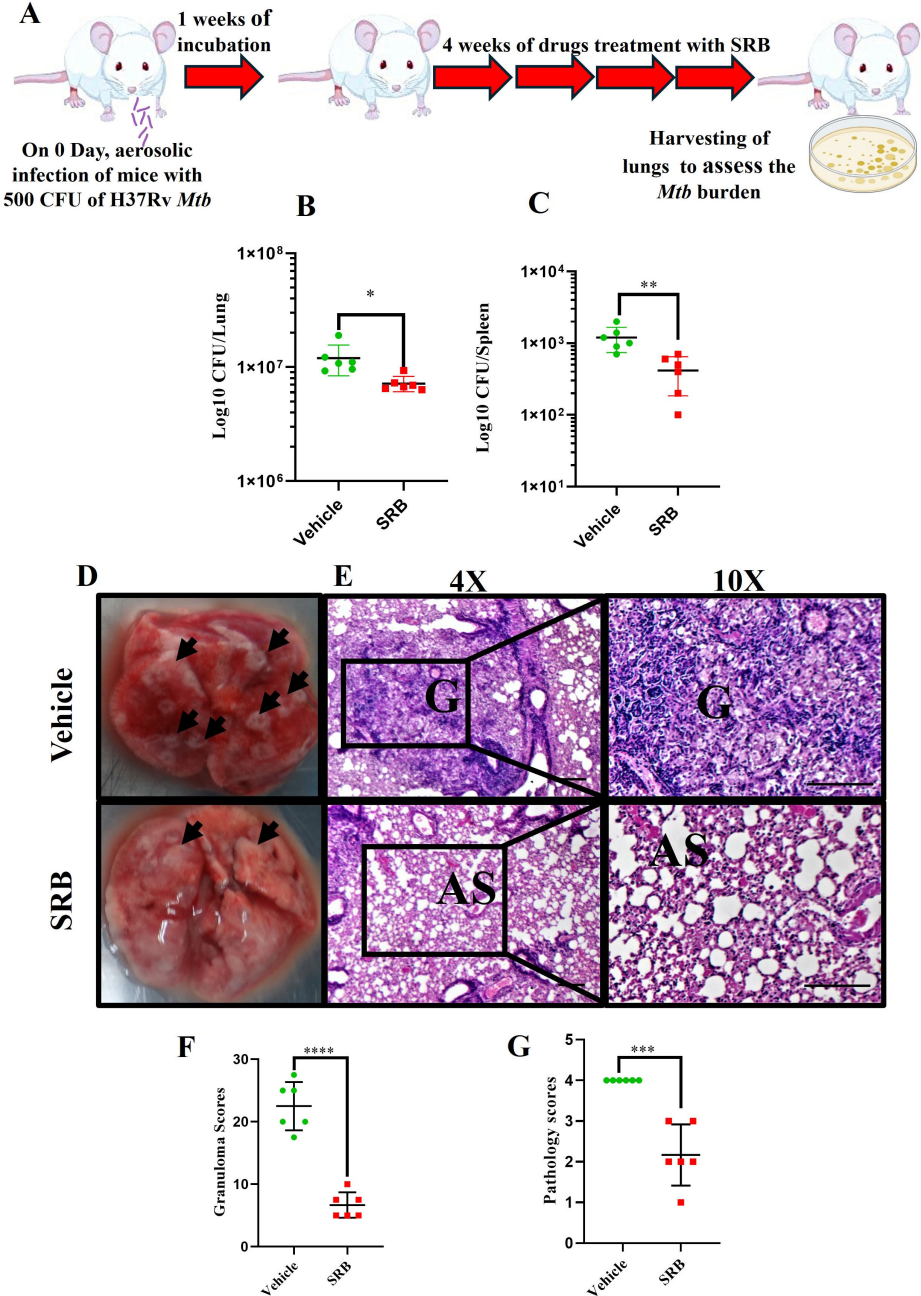
SRB treatment significantly reduces the *Mtb* burden in the lungs in an acute model infection and treatment in mice. (**A**) Schematic of *Mtb* infection and drug treatment in BALB/c mice. (**B**) Bacterial count in lungs 4 weeks post-treatment. (**C**) Bacterial count in spleens 4 weeks post-treatment. For panels B and C, the data were collected from six mice in each cohort and presented as mean ± SD in log10 CFU/lung and log10 CFU/spleen, respectively, and the statistical significance between experimental groups was determined using an unpaired Student’s *t*-test (*P < 0.05 and **P < 0.01). (**D**) Representative gross morphology of lungs from H37Rv-infected control and SRB-treated mice (4 weeks post-treatment). The black arrows on lungs correspond to tubercular granuloma. (**E**) H&E-stained representative images of histopathology of the lungs from vehicle (H37Rv-infected) control and SRB-treated groups. G, granuloma; AS, alveolar space (scale bars, 100 µm). Histopathological analysis, granuloma score (**F**), and histopathology score (**G**) for vehicle control and SRB-treated groups in the acute infection and treatment models. The data were collected from six mice in each cohort and are presented as mean ± SD, and the statistical significance between experimental groups was determined using an unpaired Student’s *t*-test (***P < 0.001; ****P < 0.0001).

Following the assessment of SRB’s antimycobacterial effects in the chronic preclinical model of infection and therapy, we looked at SRB’s antimycobacterial activity in the acute model of infection and treatment and found that the bacterial burden in the lungs and spleens of SRB-treated groups was significantly lower than that of vehicle (H37Rv-infected) control groups (Fig. 4B and C). The gross pathology (Fig. 4D) of the lungs of the vehicle control and SRB-treated groups was found to be compatible with the CFU count. The lungs of vehicle control mice showed several granulomas, whereas only a small number of granulomas were seen in the lungs of mice treated with SRB. We found moderate to minimal pathology with extensive expanses of normal alveolar space in the SRB-treated groups, while the vehicle control lungs displayed significant pathology with several small and large granulomas, alveolar consolidation, and immune cell infiltration (Fig. 4E). The granuloma and histopathology scores were found to be consistent with bacterial infection and SRB treatment (Fig. 4F and G).

### SRB treatment lowered *Mtb*-susceptible macrophage populations and upregulated Arg-positive macrophage population to heal tissue pathology

**Fig 5:**
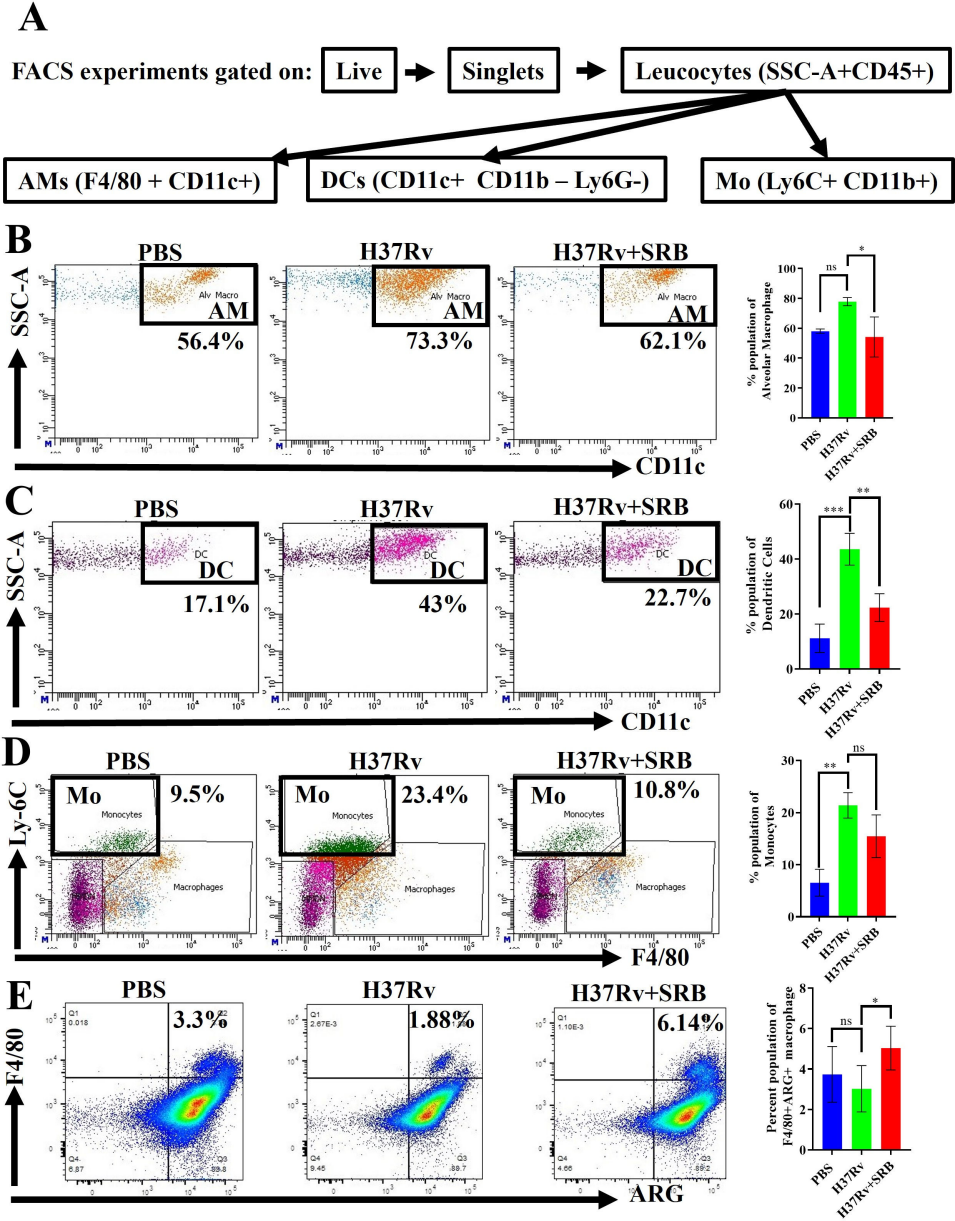
SRB treatment reduced CD11c^+^ population of macrophage (AMs and DCs) and upregulated Arg-1-positive populations of lung macrophage to heal lung pathology in the acute infection and treatment model. (**A**) General gating strategy used for flow cytometry analysis of lung immune cells obtained from PBS control, H37Rv-infected, and H37Rv+SRB-treated mice. (**B**) Identification of lung AMs (F4/80^+^ CD11c^+^), showing representative FACS plot with the percentage populations (% of parent cells acquired and bar diagram showing significant reduction in AM population in SRB-treated groups). (**C**) Identification of lung DCs (CD11c^+^ CD11b^-^ Ly6G^-^), showing representative FACS plot with the percentage populations (% of parent cells acquired) and bar diagram showing significant reduction in DC population in SRB-treated groups. (**D**) Identification of lung monocyte population (Ly6C^+^ CD11b^+^), showing representative FACS plot with the percentage populations (% of parent cells acquired) and bar diagram showing reduction in monocyte population in SRB-treated groups. (**E**) Identification of the lung Arg-1, showing representative FACS plot with the percentage populations (% of parent cells acquired) and bar diagram showing significant reduction in Arg^+^ population of macrophages in SRB-treated groups. Data information: the data were obtained from three to four biological replicates and presented as mean ± SD, and the statistical significance between experimental groups was determined by one-way ANOVA with Tukey’s multiple comparison test. *P*-values determined and represented as ns, non-significant; *P < 0.05; **P < 0.01; and ***P < 0.001.

Here, we report that the population of DCs, monocytes (Mo), and alveolar macrophages (AMs) is decreased by SRB treatment. It has been previously observed that permissive monocytes, DCs, and AMs comprise the immune cell populations most susceptible to *Mtb* infection (9, 22–25). In the current investigation, we observed that SRB-treated groups exhibited reduced populations of double-positive, F4/80^+^ CD11c^+^ macrophage phenotypes, which are indicative of resident AMs, and CD11c^+^ CD11b^-^ Ly6G^-^ DCs (Fig. 5B and C), and double-positive CD11b^+^ Ly6c^+^ monocytes (Fig. 5D). Due to their reliance on fatty acid metabolism for energy, the AM populations may transform into inflammatory foamy macrophages, which provide an environment that is conducive to *Mtb* replication and dissemination (25). There appears to be a correlation between the decrease in the AM population and the decline in bacterial load in SRB-treated groups. According to a previous study, monocyte-derived macrophages expressing high levels of CD11c are highly susceptible to *Mtb* infection (26). Additionally, we found that mice treated with SRB had a higher number of macrophages that were positive for Arg-1 (Fig. 5E) than the group infected with *Mtb*. Given the correlation between the healing of lung tissues and Arg-1-positive macrophages, our finding of their upregulation suggests that SRB has a beneficial function in both lowering the tubercular burden and the subsequent healing of tissue pathology. This is consistent with the linkage of Arg-1-positive macrophages with the repair of lung tissues (9).

## DISCUSSION

Implementation of host-directed, immunomodulation-dependent therapies to manage *Mtb* requires an understanding of the linkages and interactions that occur between *Mtb* and immune cells during the infection (9). We previously reported on the direct killing impact of multikinase inhibitor SRB on *Mtb* by the allosteric inhibition of *Mtb* ornithine acetyltransferase, a crucial enzyme of its arginine biosynthesis pathway, resulting in the inhibition of arginine production in the bacteria (11). The current study focuses on the pro-apoptotic and immunomodulatory processes that SRB triggers in the host to counteract the bacteria’s pathogenicity and ability to survive. To achieve these objectives, we carried out an infection and treatment experiment to examine the activity of SRB in chronic as well as acute *Mtb* infection and treatment of the preclinical models of mice after establishing that SRB shows no detrimental influence on mice’s health and no toxicity in essential organs such as the liver, kidney, and lungs. We observed that SRB treatment significantly reduces the *Mtb* burden in the lungs of *Mtb*-infected chronic models of infection in mice. Furthermore, we noted that SRB in combination with INH worked better than INH alone and showed maximum diminution of *Mtb* burden in infected mice after 8 weeks of treatment. An exploration of the expression of pro-apoptotic proteins by SRB treatment in a preclinical mouse model showed a decrease in phosphorylated proteins of p-AKT, p-mTORC, and p-ERK1/2, respectively, in SRB-treated groups in contrast to only *Mtb*-infected control groups. Furthermore, we found that the administration of SRB decreased the phosphorylation of Akt-1 at Ser473 and significantly lowered the phosphorylation of mTOR at Ser2488. Akt (V-akt murine thymoma viral oncogenes homolog 1) viz protein kinase B is a serine/threonine protein kinase that has essential functions in many aspects of cellular physiology including apoptosis, cell proliferation, cell migration, and controlling the intracellular signal transduction (27, 28). Previously, it was reported that *Mtb* infection induced phosphorylation of Akt at Ser473 (29), and the activation of the PI3K/AKT pathway promotes *Mtb* survival by accelerating cell cycle progression and inhibiting apoptosis (30). Activated Akt directly phosphorylates and stimulates mTORC1, blocking the formation of the unC-51-like autophagy kinase (ULK1) complex, thereby preventing the formation of autophagosomes and allowing *Mtb* to escape autophagic degradation in the host cell (30). Previously, it has been reported that SRB inhibits the activity of the mTORC1 of mTORC (31). The mTOR protein acts as a negative regulator of the autophagy pathway, which constitutes a potent target to establish new treatments for TB. It was also earlier reported that SRB inhibits the phosphorylation of the Akt, mTOR, and ERK protein, promoting JAK/STAT3 activation in various types of cancer cell lines (32, 33). In this current study, we found that the groups treated with SRB had significantly higher levels of total STAT-1 proteins. Though the function of unphosphorylated STAT-1 remains unclear, it is established that phosphorylated STAT1 regulates gene expression in macrophages infected with *Mtb* (34). Both unphosphorylated STAT1 and P-STAT1 have been identified as transcription factors in earlier research (35, 36), suggesting that they play a role in the cell death brought on by *Mtb* strain H37Ra. We further investigated the levels of pro-apoptotic proteins caspase-3 and caspase-7. We observed increased levels of cleaved caspase-3 and cleaved caspase-7 in SRB-treated groups in contrast to *Mtb*-infected control. Thus, SRB therapy promotes unphosphorylated STAT-1 while blocking the phosphorylation of Akt, mTOR, and ERK1/2, respectively. These mechanisms ultimately combine to activate caspase-3, which triggers apoptosis. Thus, SRB treatment promotes apoptosis in *Mtb*-infected and -uninfected THP-1 cells. Concurrently, SRB treatment reduces the infection-associated necrosis in *Mtb*-infected human monocyte-derived macrophages.

The role of apoptosis in the host’s defense against intracellular infections is now increasingly acknowledged, and apoptosis has long been proposed as a mechanism to control TB (13, 37–40). On the contrary, necrosis increases the burden and spread of bacteria in *Mtb*-infected macrophages and animals (41–45). Because necrosis contributes to both tissue damage and bacterial dissemination, it is a possible target for intervention in the pathophysiology of TB (38, 46). *Mtb* resists apoptosis and SRB treatment increases apoptotic activities, indicating that *Mtb* and SRB have antagonistic effects on apoptotic processes. Furthermore, we observed that SRB inhibits necrosis while *Mtb* encourages it; in this case, *Mtb* and SRB also behave antagonistically toward necrosis-related activities. In conclusion, we describe here the special ability of SRB to enhance apoptosis and prevent necrosis in THP-1 cells infected with *Mtb.* This characteristic of SRB is highly beneficial in preventing the spread and transmission of TB and is consistent with a number of studies in the past on *Mtb* (47–51).

Previous studies have also shown that immunosuppressive cytokines, including IL-10 and TGF-β, as well as FoxP3+ Treg cells, are more prevalent in people with active TB (20, 21). Our findings are consistent with these observations. We also demonstrate a significant upregulation of pro-inflammatory cytokines and downregulation of immunosuppressive cytokines. Following the assessment of SRB’s antimycobacterial effects in the chronic preclinical model of infection and therapy, we looked at SRB’s antimycobacterial activity in the acute model of infection and treatment. We noted the bactericidal activity of SRB and found that the bacterial burden in the lungs and spleens of SRB-treated groups was significantly lower than that of *Mtb* control groups. Here, we report that the population of DCs, monocytes (Mo), and AMs decreased by SRB treatment. It has been previously observed that permissive monocytes, DCs, and AMs comprise the immune cell population most susceptible to *Mtb* infection (**22–25**). The presence of tissue repair-associated macrophage phenotypes with variable activation is indicated by the expression of the proteins Ym1 (chitinase 3-like 3) and Arg-1 in mice (52–56). Here, we found that, in the acute model of infection and treatment, mice treated with SRB had a higher number of macrophages that were positive for Arg-1 than the group infected with *Mtb*. In conclusion, we found that SRB plays a beneficial function in both lowering the tubercular burden and the subsequent healing of tissue pathologies because Arg-1-positive macrophages are linked to the repair of lung tissues. SRB not only lowers the tubercular load but also augments the tissue repair process. Thus, the induction of SRB augurs well for anti-tubercular therapeutics.
